# Development and validation of a condition-specific diary to measure severity, bothersomeness and impact on daily activities for patients with acute urinary tract infection in primary care

**DOI:** 10.1186/s12955-017-0629-5

**Published:** 2017-03-24

**Authors:** Anne Holm, Gloria Cordoba, Volkert Siersma, John Brodersen

**Affiliations:** Research Unit for General Practice and Section of General Practice, Department of Public Health, University of Copenhagen, Øster Farimagsgade 5, PO box 2099, 1014 Copenhagen, Denmark

**Keywords:** Urinary tract infections, Cystitis, Validation studies, Psychometrics, Item-response theory, Rasch analysis, Patient-reported outcomes, Patient-reported outcome measures, PROM, Primary care

## Abstract

**Background:**

Urinary tract infection (UTI) is a common condition in primary care. Patient-reported outcome measures (PROMs) are crucial in the evaluation of interventions to improve diagnosis, treatment and prognosis of UTI. The aim of this study was to identify an existing condition-specific PROM to measure symptom severity, bothersomeness and impact on daily activities for adult patients with suspected urinary tract infection in primary care; or, in the absence of such a PROM, to test items identified from existing PROMs for coverage and relevance in single and group interviews and to psychometrically validate the resulting PROM.

**Methods:**

The literature was searched for existing PROMs covering the three domains. Items from the identified PROMs were tested in single and group interviews. The resulting symptom diary was psychometrically validated using the partial credit Rasch model for polytomous items in a cohort of 451 women participating in two studies regarding UTI.

**Results:**

No existing PROM fulfilled the inclusion criteria. Content validation resulted in one domain concerning symptom severity (18 items), one concerning bothersomeness (18 items), and one concerning impact on daily activities (7 items). Psychometrical validation resulted in four dimensions in each of the first two domains and one dimension in the third domain.

**Conclusions:**

Domains were not unidimensional, which meant that we identified dimensions of patient-experienced UTI that differed substantially from those previously found. We recommend that future studies on UTI, in which PROMs are to be used, should ensure high content validity of their outcome measures and unidimensionality of the included dimensions.

**Electronic supplementary material:**

The online version of this article (doi:10.1186/s12955-017-0629-5) contains supplementary material, which is available to authorized users.

## Background

Urinary tract infection (UTI) is a common condition and accounts for about 2% of consultations in general practice in Denmark [1]. It mainly affects women, one in every two women experiences a UTI at least once in her life-time [2]. Symptoms of UTI are known to be painful and bothersome, impacting quality of life [3–6]. In addition to the symptoms experienced by the patient, laboratory confirmation of a significant amount of bacteria in the urine is required for the diagnosis of a clinically relevant UTI [7]. Patient-reported outcome measures (PROMs) are important both for the evaluation of the extent to which an intervention can improve the diagnosis and treatment of UTI, and for following the patient’s experience of symptoms and recovery. A PROM should be face and content validated to ensure that its items are relevant and covering for the construct that is to be measured. Moreover, items and instructions in the PROM should be clear and understandable for the target population [8–10]. If the PROM encompasses domains of items, then these should be psychometrically validated in a larger sample of the target population using item response theory (IRT) models to ensure unidimensionality of the domains allowing for sum scores [11]. When items in a domain fit IRT Rasch models, invariant measurement is achieved [12–16]. A number of PROMs exist, but to our knowledge none of them have been tested for both content validity and unidimensionality of domains using IRT models [6, 17–19].

The aims of this study were to 1) Perform a literature search to identify an existing condition-specific PROM to measure symptom severity, bothersomeness and impact on daily activities over time for adult patients with uncomplicated and complicated UTI in primary care; or 2) in the absence of such a PROM, to test items identified from existing PROMs for relevance in single and group interviews with patients who had experienced UTI; and 3) to psychometrically validate the resulting PROM using Rasch models.

## Methods

### Aim 1: literature search for existing PROMs

We searched Medline and Embase for development and validation studies published before September 2014 in English, Swedish, Danish or Norwegian. Combinations of the words “urinary tract infection”, “cystitis”, “patient-reported outcome measure”, “psychometrics”, “PROM”, “instrument”, “validation” and “scale” were used.

#### Inclusion criteria

PROM development and validation studies performed in primary care or a comparable setting investigating adult patients with symptoms of UTI including the three domains: Symptom severity, symptoms bothersomeness and impact on daily activities and reporting a sufficient content validation involving either single or groups interviews to ensure coverage and relevance of items and a sufficient content validation using IRT models and analysis of differential item functioning (DIF).

### Aim 2: face and content validity

#### Overview of content validation procedure

The process of content validation involved two primary elements: 1) Item generation and construction of a draft PROM, 2) single and group interviews with members of the target population.

#### Item generation

Items relevant to the three domains were selected from existing PROMs identified in the literature search. To narrow down the initial pool of items, the items that had proved most predictive of confirmed UTI in previous research were selected and some items were modified based on clinical experience [20, 21]. Double-barreled items (For example “pain or burning when passing urine”) were split into two individual items. The resulting draft version of the PROM was converted into a 7-day symptom diary, one of several types of PROMs.

#### Group interviews

Group interviews were aimed at expanding our knowledge on symptoms experienced by patients with UTI, their bothersomeness and impact on daily activities. The method of group interviews was chosen to ensure a dynamic generation of new items and an open discussion about the content and layout of the diary [22]. The participants were encouraged to talk about their experience of having UTI using open-ended questions. When they had no more new symptoms or activities to add, the draft version of the diary was presented. The participants completed the draft version of the diary and it was corrected according to their suggestions. Participants were recruited from a general practice, an elderly activity center and the researchers’ network. The group interviews took about two hours and were audio recorded; the recordings were used to analyze the interviews and change the draft version of the diary.

#### Single interviews

The purpose of single interviews was to ensure relevance, coverage and understandability of the diary. The participant firstly told about his or her experience of the UTI. Afterwards, the participant was told to complete the diary and comment on relevance, coverage and understandability. Female participants were recruited from the researchers’ network and male participants were recruited at a urological department. Single interviews lasted about 30 min.

### Aim 3: psychometric validation

#### Patient recruitment

Patients with symptoms of UTI participating in two ongoing studies [23, 24] were asked to complete the diary after seeing their general practitioner. The diary was handed out in the consultation and the participant returned it by post using a prepaid reply envelope. We used text-message reminders and telephone calls to remind patients to complete and return the diary.

#### Statistical analysis: Rasch analysis

The responses were analyzed using the partial credit Rasch model for polytomous items [25, 26]. If an item shows misfit to a Rasch model, it indicates that the item does not belong to the same theoretical dimension. We tested the three domains for unidimensionality. If items showed misfit we tested alternative configurations of items based on clinical, empirical or theoretical relations between symptoms rather than results from analyses. The resulting dimensions were tested for DIF. DIF indicates that other factors, such as age, affect the responses to a specific item, causing the scale to behave differently in the different subgroups [27, 28]. Finally, we tested for local dependency (LD) to evaluate whether individual items within the resulting dimensions were so closely linked that they, to some extent, were measuring the same nuances of the construct. If two items have high local dependency, they nearly correspond to a single item. Since individual symptoms are known to have poor predictive value for confirmed UTI, we did not test for discriminative ability of the identified dimensions [20, 21]. If an item did not fit any dimensions it was kept in the final questionnaire if it had high content validity.

#### Data management and statistics

The psychometric properties of the involved scales were tested for unidimensionality, homogeneity and DIF in relation to age, study group, and confirmed UTI, by using likelihood ratio tests on appropriately conditioned Rasch models [29]. Confirmed UTI was defined as having significant growth of uropathogens in a reference culture. The patient was not aware of the result at the time of completing the diary. The reliability of the dimensions was examined using Cronbach’s alpha (Table 2). Statistical analyses were performed in DIGRAM [30]. To adjust for multiple testing the false discovery rate was fixed at 5% for each set of analyses using the Benjamini-Hochberg method [31].

## Results

### Aim 1: literature search for existing PROMs

No PROMs were identified measuring all three domains: symptom severity, bothersomeness and impact on daily activities. We identified four development and validation studies for patients with symptoms of UTI [6, 17–19]. None of these studies described the use of single or group interviews to ensure content validity. All of them were statistically validated, but only one study tested for unidimensionality using IRT but not for DIF [17]. The identified studies are listed in Additional file 1.

### Aim 2: face and content validity

#### Item generation

**Fig. 1:**
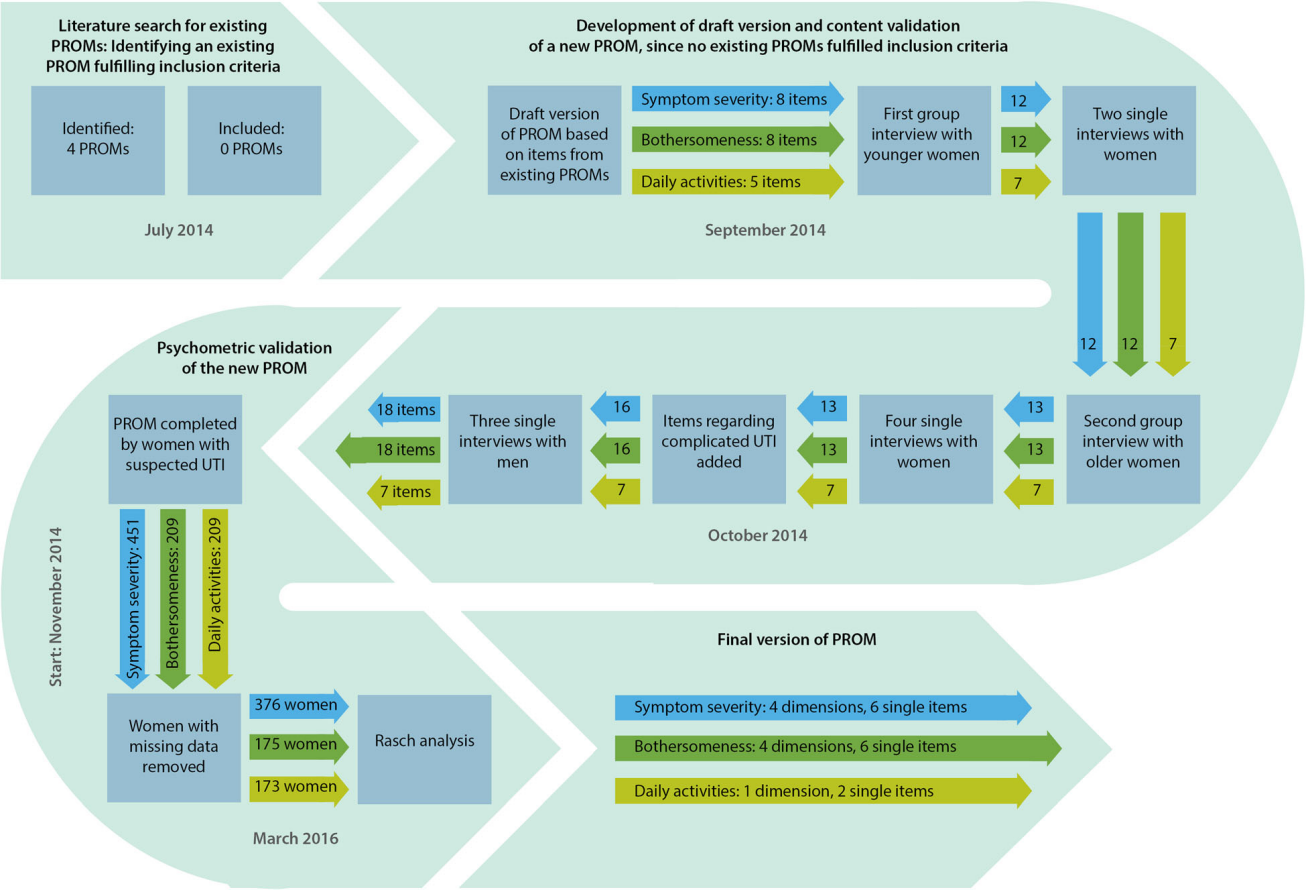
Content and psychometric validation process and inclusion of items. Legend: UTI = Urinary tract infection

The first draft version of the diary contained eight items regarding symptom severity, eight items regarding symptom bothersomeness and five items regarding daily activities (Fig. 1). Four response categories to these 21 items were drafted: 0 (none), 1 (a little), 2 (some) and 3 (a lot). Before interviewing men, six items regarding complicated UTI were added.

#### Single and group interviews

Two group interviews were conducted: one with four women aged 29–63 (six invited) and one with seven women from 70 to 89 (seven invited). The first group was in the latter part of the interview presented to a draft questionnaire including 21 items (Fig. 1). In the first group interview twelve new items (four on symptom severity, four on symptom bothersomeness and four on activities) were generated (Fig. 1). None of the symptoms in the draft version was considered irrelevant, but two items regarding activities were discarded (ability to concentrate and spare time activities). In the second group interview with elderly women, almost all of the same symptoms were repeated but two new items were generated: Severity and bothersomeness of feeling unwell. The elderly women had more problems identifying individual activities that were impacted when they had UTI but did not find the activities from the draft version irrelevant. They also found the diary quite long and repetitive to complete. They could, however, accept completing the items in all three scales in a single day and just the items in one scale on the following days. Both groups found the response categories sufficient and used all four options when completing the diary. We had planned to perform a group interview with men as well, but recruitment proved so difficult that we decided to conduct single interviews with men.

Six single interviews with women were performed – two after the changes following the first group interview and four after the second group interview. The result of these single interviews was minor corrections to phrasing and layout. No new items were generated in the single interviews with women.

Three men were interviewed; one interview was performed in person and two were conducted over the telephone after the diary had been sent in the post. All three men had experienced having both cystitis and pyelonephritis. Their vocabulary for describing their symptoms was different, but they found the vocabulary in the diary understandable and the items relevant. The first interview resulted in four new items related to complicated UTI. No additional items were generated in either of the other two interviews.

After four single interviews with women and two with men without any new items, we concluded that data saturation had been reached.

The result of the qualitative evaluation was three domains: one for symptom severity containing 18 items, one for symptom bothersomeness containing 18 items and one for impact on daily activities containing seven items. The process and result of the content validation can be seen in Fig. 1.

### Aim 3: psychometric validation

#### Recruitment of patients

We included data on 451 female patients consulting their general practitioner with at least one UTI symptom. 209 of the women completed the full questionnaire regarding symptom severity, bothersomeness and daily activities. The remaining 242 only completed the items regarding symptom severity. Response rates in the two studies were 86 and 78%. We included age, job status, if the patient had confirmed UTI and which study she participated in as covariates. Inclusion of patients can be seen in Fig. 1 and characteristics of included patients in Table 1.

**Table 1:** Characteristics of patients included in psychometric validation

	Study 1 (all domains) n (%)	Study 2 (symptom severity only) n (%)
Age group		
0–24 years	17 (9.8)	20 (9.9)
25–34 years	29 (16.7)	23 (11.4)
35–54 years	50 (28.7)	73 (36.1)
55–69 years	48 (27.6)	50 (24.8)
70 years or more	30 (17.2)	36 (17.8)
Job status		
Employed	90 (51.7)	No data
Under education	21 (12.1)	No data
Job seeking	7 (4.0)	No data
Retired or otherwise not job seeking	56 (32.2)	No data
Confirmed UTI (growth in urine culture)		
Confirmed UTI	133 (76.4)	118 (58.4)
No UTI	41 (23.6)	84 (41.6)

Numbers in this table refer to the 376 women used to analyze domain S (symptom severity). Domain B (bothersomeenss, *n* = 175) and D (daily activities, *n* = 173)) were validated with data from study 1

#### Rasch analysis

None of the three domains revealed unidimensionality in the initial Rasch analyses. Subsequent empirical analysis of the three domains revealed nine new dimensions covering symptom severity, symptom bothersomeness and impact on daily activities. 14 single items did not fit the dimensions, but could not be excluded from the diary without compromising content validity. The overall fit of the nine dimensions can be seen in Table 2 and the fit of individual items in Table 3.

**Table 2:** Initial three domains and overall fit statistics

	Dimension (n items)	CLR *χ* ^2^	DF	*P*	Chronbach alpha
Symptoms	Dysuria (3)	12.1	8	0.146	0.554
	Frequency (4)	17.2	11	0.102	0.823
	Lower back (2)	8.5	5	0.132	0.938
	General (3)	8.4	8	0.392	0.735
Bothersomeness	Dysuria (3)	6.2	8	0.629	0.574
	Frequency (4)	21.1	11	0.032	0.839
	Lower back (2)	0.3	5	0.998	0.930
	General (3)	4.2	8	0.840	0.716
Daily Activities	Daily activities (5)	22.6	14	0.067	0.888

Initial three domains and overall fit statistics A NON-significant *P*-value for CLR *χ* ^2^ suggests a good fit to the unidimensional model. A high Chronbach alpha suggests the dimension has internal consistency. *CLR χ* ^2^ = conditional likelihood chi-square, *DF* degrees of freedom

**Table 3:** Fit statistics of individual items

Final dimension	Item number	Item	Item rest-score		SD	*P*
			observed	expected		
Symptom severity						
Dysuria	1	Pain on urination	0.380	0.367	0.046	0.765
	2	Difficult to empty bladder	0.370	0.375	0.047	0.928
	3	Uncomfortable pressure around the bladder	0.375	0.365	0.046	0.831
Frequency	4	Frequent urination - daytime	0.704	0.679	0.034	0.474
	5	Increased urge for urination	0.682	0.682	0.034	0.999
	6	Has to hurry to the toilet	0.737	0.690	0.030	0.118
	7	Incontinence	0.672	0.704	0.032	0.314
Lower back	8	Pain in lower back	0.957	0.957	0.011	0.993
	9	Uncomfortable pressure in lower back	0.957	0.957	0.011	0.993
General	10	Feeling unwell	0.663	0.651	0.041	0.770
	11	Fever	0.705	0.669	0.044	0.422
	12	Shivering	0.629	0.672	0.043	0.327
Single items	13	Burning sensation on urination	–	–	–	–
	14	Smelly urine	–	–	–	–
	15	Urine changed appearance	–	–	–	–
	16	Blood in urine	–	–	–	–
	17	Frequent urination - nighttime	–	–	–	–
	18	Pain around the bladder	–	–	–	–
Symptom bothersomeness						
Dysuria	19	Pain on urination	0.396	0.365	0.067	0.650
	20	Difficulty emptying bladder	0.385	0.403	0.067	0.790
	21	Uncomfortable pressure around the bladder	0.397	0.390	0.066	0.917
Frequency	22	Frequent urination - daytime	0.663	0.690	0.045	0.551
	23	Increased urge to urinate	0.699	0.693	0.046	0.888
	24	Has to hurry to the toilet	0.758	0.704	0.043	0.217
	25	Incontinence	0.724	0.727	0.046	0.946
Lower back	26	Pain in lower back	0.966	0.967	0.014	0.987
	27	Uncomfortable pressure in lower back	0.966	0.967	0.014	0.987
General	28	Feeling unwell	0.692	0.707	0.056	0.792
	29	Fever	0.766	0.694	0.059	0.223
	30	Shivering	0.671	0.714	0.059	0.457
Single items	31	Burning sensation on urination	–	–	–	–
	32	Smelly urine	–	–	–	–
	33	Urine changed appearance	–	–	–	–
	34	Blood in urine	–	–	–	–
	35	Frequent urination - nighttime	–	–	–	–
	36	Pain around the bladder	–	–	–	–
Daily activities						
Daily activities	37	Work	0.861	0.762	0.039	0.010*
	38	Social activities	0.811	0.768	0.038	0.251
	39	Exercise	0.758	0.775	0.036	0.652
	40	Cycling	0.620	0.777	0.042	0.000**
	41	Tasks in the home	0.792	0.767	0.040	0.538
Single items	42	Sleep				
	43	Sex				

A non-significant *P*-value suggests a good fit to the unidimensional model of the individual items. Critical levels adjusted by the Benjamini-Hochberg procedure: * < 5% FDR, ** < 1% FDR

#### Symptom severity (Domain S)

We suggested domain S to have a dimension regarding frequency and a dimension regarding pain (dysuria). Four symptom-items fitted the Rasch model in the frequency-dimension: Frequent urination – daytime, Increased urge for urination, Having to hurry to the toilet and Incontinence. Four combinations of items showed LD: Frequency and Urge, Frequency and Incontinence, Incontinence and Urge, and Incontinence and Having to hurry to the toilet. Three items fitted the Rasch model in the pain-dimension (Pain on urination, Difficult to empty bladder and Uncomfortable pressure around the bladder). One combination – Pain around the bladder and Difficult to empty bladder – showed LD. We suggested a dimension regarding symptoms from the lower back, and two items fitted this with no LD. Finally, we tested a dimension regarding general symptoms, which encompassed three items and had a high fit to the model and no LD. None of the final four dimensions showed DIF. Six items regarding symptom severity did not fit a dimension.

#### Symptom bothersomeness (Domain B)

Since the bothersomeness domain contained the same items as the symptom domain, but asked about bothersomeness instead of severity, we tested the dimensions identified in the analysis of domain S. All four dimensions fitted the model and showed no DIF. There were only two combinations of symptoms in the frequency dimension showing LD: Frequency and Urge, and Incontinence and Having to hurry to the toilet.

#### Impact on daily activities (Domain D)

The daily activities domain showed unidimensionality and no DIF if two items (Sleep and Sex) were removed. These two items were removed because they were related to nighttime, which the other items were not. These two items did not compose a separate dimension together. The item “Cycling” showed a low fit to the dimension, but removing it did not improve overall fit, so we decided to keep it in the dimension. The final dimension showed high levels of LD; only three combinations did not have LD: Work and Exercise, Social activities and Exercise, and Social activities and Domestic duties.

## Discussion

This study resulted in a substantially new symptom diary for patients with symptoms of UTI with high content validity and adequate psychometric properties, comprising four dimensions of symptom severity and bothersomeness – dysuria, frequency, lower back symptoms and general symptoms – as well as one dimension of impact on daily activities. This is to our knowledge the first symptom diary regarding UTI to have been both content and psychometrically validated.

### Strengths and limitations of this study

The diary was developed through interviews with patients attending general practice, thus yielding high content validity for patients in this setting. The domains were psychometrically analyzed using a large cohort obtained through two different studies. The psychometric validation ensured unidimensionality of the scales within the three domains and no DIF. We found corresponding scales in the symptom severity and symptom bothersomeness domains, suggesting the scales to be a solid finding.

It is a limitation in this study that we were unable to recruit men for a group interview or for the psychometrical validation. However, single interviews with men showed good relevance and coverage of the identified items and the items generated in the interviews with men were not gender-specific, but related to complicated UTI. In the psychometrical validation, we did not have sufficient sociodemographic data to include covariates, such as job status and education, in all analyses. This does not compromise the identified domains, but we do not have data to confirm whether any of the items possessed DIF in relation to sociodemographics. Another weakness is the high level of LD in the scale regarding daily activities. However, this finding corresponds with data from our second group interview, where participants stated that all activities were equally affected when they had UTI. This PROM is for research purposes and the fit-statistics indicate it should not be used for individual patients.

### Findings in relation to other studies

Previous instruments regarding symptoms of UTI have also covered aspects such as frequency and dysuria [18]. However, our content validation process showed that patients do not see frequency as a uniform aspect that can be scored in a single item, but as a group of symptoms and experiences of having to hurry to the toilet, having to void often in both the daytime and the nighttime and having incontinence problems. The psychometric validation showed that most of these items – but not all – were part of the same construct. Urgency, which is usually investigated separately, turned out to be part of the frequency scale. The term dysuria was even more differently perceived by patients than by us, the clinicians. The content validation resulted in several new items dealing with different aspects of pain, since the term “pain” turned out to be too broad a concept. In the psychometric validation we found a three-item dimension comprising “pain on urination”, “difficulties emptying the bladder” and “uncomfortable pressure around the bladder”; but the items “a burning sensation on urination” and “pain around the bladder” were not part of this dimension and the patients must have perceived these as fundamentally different symptoms.

### Unanswered questions and future research

This study demonstrates that patient-experienced symptoms differ from the ways in which professionals perceive them as has been previously shown [4]. It indicates that patient interviews with the target population should always be conducted before introducing a new instrument. The study has identified new dimensions of patient-experienced UTI that differ, in terms of content, from those previously been found. The symptom diary is a robust instrument when used in studies investigating women with UTI symptoms in general practice, but we do not have sufficient data to determine whether it could be used in a male population. Before using it in a study on male patients, we would suggest performing a psychometric validation on men. We recommend that future studies on UTI, in which PROMs are to be used, should ensure high content validity of their outcome measures and unidimensionality of the included dimensions.

## Conclusions

Several instruments have been validated to measure symptoms in patients with suspected UTI. Items and dimensions are usually generated by the researcher and statistical validation does not test for unidimensionality, but assumes, rather, that each item represents a different feature of the same construct. This study has content and psychometrically validated a new symptom diary for UTI, identifying nine unidimensional scales measuring different constructs of symptom severity, bothersomeness and impact on daily activities in patients with UTI. These scales differ substantially from those previously described in the scientific literature.

## Additional file

Additional file 1: Identified PROMs. (DOCX 27 kb)

## Abbreviations

DIF: Differential item functioning
IRT: Item response theory
LD: Local dependency
PROM: Patient-reported outcome measure
UTI: Urinary tract infection
