# Supplementary material for: Development and validation of a condition-specific diary to measure severity, bothersomeness and impact on daily activities for patients with acute urinary tract infection in primary care
Source: Health Qual Life Outcomes. 2017 Mar 24;15:57. doi: 10.1186/s12955-017-0629-5 (PMC5366156; doi:10.1186/s12955-017-0629-5)
Supplement: Additional file 1: — Identified PROMs. (DOCX 27 kb) [file 12955_2017_629_MOESM1_ESM.docx]

# Additional file 1 Identified PROMs

| **Study** | **Patients** | **Domains** | **Content validity** | **Psychometric validity** | **Items** | **Included in first draft** | **Included after first group interview** | **Included before interviewing men** | **Not included** |
| --- | --- | --- | --- | --- | --- | --- | --- | --- | --- |
| Alidjanov JF, New self-reporting questionnaire to assess urinary tract infections and differential diagnosis: acute cystitis symptom score [1]. | Female patients | Typical symptoms, differential diagnosis symptoms (relevant to severity and bothersomeness) | No interviews described | No test for unidimensionality or differential item functioning | Severity (typical symptoms) |  |  |  |  |
|  |  |  |  |  | Frequency | X |  |  |  |
|  |  |  |  |  | Urgency | X |  |  |  |
|  |  |  |  |  | Painful urination | X |  |  |  |
|  |  |  |  |  | Incomplete emptying |  | X |  |  |
|  |  |  |  |  | Suprapubic pain | X |  |  |  |
|  |  |  |  |  | Hematuria | X |  |  |  |
|  |  |  |  |  | Severity (differential diagnosis) |  |  |  |  |
|  |  |  |  |  | Flank pain |  |  | x |  |
|  |  |  |  |  | Vaginal discharge |  |  |  | X |
|  |  |  |  |  | Urethral discharge |  |  |  | X |
|  |  |  |  |  | Feeling of a fever |  |  | X |  |
|  |  |  |  |  | Hyperthermia |  |  |  | X |
| Clayson D .Validation of a patient-administered questionnaire to measure the severity and bothersomeness of lower urinary tract symptoms in uncomplicated urinary tract infection (UTI): the UTI Symptom Assessment questionnaire [2]. | Female patients | Severity and bothersomeness | No interviews described | No test for unidimensionality or differential item functioning | Frequency of urination (going to the toilet very often) | X |  |  |  |
|  |  |  |  |  | Urgency of urination (a strong and uncontrollable urge to pass urine) | X |  |  |  |
|  |  |  |  |  | Pain or burning when passing urine | X |  |  |  |
|  |  |  |  |  | Not being able to empty your bladder completely/passing only small amounts of urine |  | X |  |  |
|  |  |  |  |  | Pain or uncomfortable pressure in the lower abdomen/pelvic area caused by your urinary tract infection | X |  |  |  |
|  |  |  |  |  | Low back pain caused by your urinary tract infection |  |  | X |  |
|  |  |  |  |  | Blood in your urine | X |  |  |  |
| Wild DJ. Validation of a patient-administered questionnaire to measure the activity impairment experienced by women with uncomplicated urinary tract infection: the Activity Impairment Assessment (AIA) [3]. | Female patients | Activity Impairment | No interviews described | Unidimentionality tested but not differential item functioning | Cut down on time at work | X |  |  |  |
|  |  |  |  |  | Accomplished less |  |  |  | X |
|  |  |  |  |  | Limited in kind of work | X |  |  |  |
|  |  |  |  |  | Difficulty performing work | X |  |  |  |
|  |  |  |  |  | Interfered with social activity | X |  |  |  |
| Colgan . Survey of symptom burden in  women with uncomplicated  urinary tract infections [4]. | Women 18-55 | Bothersomeness | No interviews described | None | Frequent urination | X |  |  |  |
|  |  |  |  |  | Urgency in urination | X |  |  |  |
|  |  |  |  |  | Pain during urination (dysuria) | X |  |  |  |
|  |  |  |  |  | Inability to empty bladder |  | X |  |  |
|  |  |  |  |  | Pain/pressure in lower stomach | X |  |  |  |
|  |  |  |  |  | Lower back pain |  |  | X |  |
|  |  |  |  |  | Urine leakage |  | X |  |  |

[1] J. F. Alidjanov *et al.*, “New self-reporting questionnaire to assess urinary tract infections and differential diagnosis: Acute cystitis symptom score,” *Urol. Int.*, vol. 92, pp. 230–236, 2014.

[2] D. Clayson, D. Wild, H. Doll, K. Keating, and K. Gondek, “Validation of a patient-administered questionnaire to measure the severity and bothersomeness of lower urinary tract symptoms in uncomplicated urinary tract infection (UTI): the UTI Symptom Assessment questionnaire.,” *BJU Int.*, vol. 96, no. 3, pp. 350–9, Aug. 2005.

[3] D. Wild and D. Clayson, “Validation of a patient-administered questionnaire to measure the activity impairment experienced by women with uncomplicated urinary tract infection: the Activity Impairment,” *Heal. Qual. Life Outcomes*, vol. 3, no. 42, 2005.

[4] R. Colgan, K. Keating, and M. Dougouih, “Survey of symptom burden in women with uncomplicated urinary tract infections.,” *Clin. Drug Investig.*, vol. 24, no. 1, pp. 55–60, Jan. 2004.
